# Growing *Burkholderia pseudomallei* in Biofilm Stimulating Conditions Significantly Induces Antimicrobial Resistance

**DOI:** 10.1371/journal.pone.0009196

**Published:** 2010-02-12

**Authors:** Chakrit Sawasdidoln, Suwimol Taweechaisupapong, Rasana W. Sermswan, Unchalee Tattawasart, Sumalee Tungpradabkul, Surasakdi Wongratanacheewin

**Affiliations:** 1 Melioidosis Research Center, KhonKaen University, KhonKaen, Thailand; 2 Department of Microbiology, KhonKaen University, KhonKaen, Thailand; 3 Department of Biochemistry, KhonKaen University, KhonKaen, Thailand; 4 Department of Oral Diagnosis, KhonKaen University, KhonKaen, Thailand; 5 Department of Biochemistry, Mahidol University, Bangkok, Thailand; Charité-Universitätsmedizin Berlin, Germany

## Abstract

**Background:**

*Burkholderia pseudomallei*, a Gram-negative bacterium that causes melioidosis, was reported to produce biofilm. As the disease causes high relapse rate when compared to other bacterial infections, it therefore might be due to the reactivation of the biofilm forming bacteria which also provided resistance to antimicrobial agents. However, the mechanism on how biofilm can provide tolerance to antimicrobials is still unclear.

**Methodology/Principal Findings:**

The change in resistance of *B. pseudomallei* to doxycycline, ceftazidime, imipenem, and trimethoprim/sulfamethoxazole during biofilm formation were measured as minimum biofilm elimination concentration (MBEC) in 50 soil and clinical isolates and also in capsule, flagellin, LPS and biofilm mutants. Almost all planktonic isolates were susceptible to all agents studied. In contrast, when they were grown in the condition that induced biofilm formation, they were markedly resistant to all antimicrobial agents even though the amount of biofilm production was not the same. The capsule and O-side chains of LPS mutants had no effect on biofilm formation whereas the flagellin-defective mutant markedly reduced in biofilm production. No alteration of LPS profiles was observed when susceptible form was changed to resistance. The higher amount of *N*-acyl homoserine lactones (AHLs) was detected in the high biofilm-producing isolates. Interestingly, the biofilm mutant which produced a very low amount of biofilm and was sensitive to antimicrobial agents significantly resisted those agents when grown in biofilm inducing condition.

**Conclusions/Significance:**

The possible drug resistance mechanism of biofilm mutants and other isolates is not by having biofilm but rather from some factors that up-regulated when biofilm formation genes were stimulated. The understanding of genes related to this situation may lead us to prevent *B. pseudomallei* biofilms leading to the relapse of melioidosis.

## Introduction

Melioidosis is the disease caused by gram negative bacterium, *Burkholderia pseudomallei*. The disease is endemic in Southeast Asia and Northern Australia. Clinical manifestations can be varied from acute infection, chronic localized pathologic symptoms to latent infection that may reactivate decades later. In Thailand, the disease accounts for 20% of all community-acquired septicemias and the most common cause of the high mortality is septic shock [1], [2]. *B. pseudomallei* is intrinsically resistant to many antimicrobial agents including first and second generations of cephalosporins, penicillins, macrolides, colistin, rifamycins, and aminoglycosides [3], [4]. Ceftazidime (CTZ), the carbapenems such as imipenem and meropenem, and to a lesser degree amoxicillin-clavulanate, remain the backbone of current initial or intensive phase melioidosis treatment. Resistance to CTZ and imipenem (IMN) is rare. The current standard treatment with agents to which *B. pseudomallei* is susceptible requires 2–4 weeks of parenteral therapy e.g. with CTZ as initial intensive therapy, followed by 3–6 months of oral eradication therapy e.g. with trimethoprim/sulfamethoxazole (TMP/SMX), doxycycline (DOX), chloramphenicol or a combination therapy. Although, CTZ is the drug of choice that is the most effective for treatment of severe melioidosis, the mortality rate in treated patients has been found to be more than 40% [2]. *B. pseudomallei* was reported to form biofilms and microcolonies [5]. The capacity of *B. pseudomallei* to produce biofilm varied in quantity in each isolate and there was no correlation between biofilm production and source of isolation, including the virulence of bacteria [6]. It was found that biofilm bacteria can be up to 1,000 times more resistant to antimicrobial agents than their free-living (planktonic) counterpart [7]. Moreover *B. pseudomallei* was reported to cause very high relapse rate compared to other bacterial infection [8]. The relapse might be due to reactivation of the biofilm forming bacteria that made them resist to antimicrobials. The role of biofilm in the susceptibility to antimicrobials in the same planktonic and biofilm strain of *B. pseudomallei* was never been reported. Therefore the study of the role of biofilm in antimicrobial resistance in *B. pseudomallei* is needed.

Lipopolysaccharide (LPS) of *B. pseudomallei* seems to differ in several aspects from the LPS of other gram-negative bacteria and was found to be largely conserved across this species [9]. LPS profiling of 1,327 *B. pseudomallei* isolates are mostly (97%) smooth type A LPS that possess different ladder profiles from the two less frequent types, smooth type B and rough type. Interestingly, the latter were found more in clinical than environmental isolates and also were more often associated with relapse than with primary infection [10]. Among these 3 types, type A produced the lowest amount of biofilm [10]. Apart from being immunogenic and virulence factors, LPS also acts as a permeability barrier at bacterial surfaces, particularly to hydrophobic agents [11]. The modification of LPS upon exposure to some antimicrobial agents and the defect in LPS structure from mutations have been reported to cause the loss of its resistance [12]. In *B. pseudomallei*, the direct relationship between the differentiation of LPS phenotypes and the susceptibility to antimicrobial agents, including the alterations of LPS after the formation of biofilms or exposure to antimicrobial agents are still unknown.

Quorum sensing (QS) is also one of the putative virulence factors in *B. pseudomallei* [13]. In gram-negative bacteria, it is a cell-density-dependent communication system that uses *N*-acyl homoserine lactones (AHLs) for the coordination of gene expression. Bacterial biofilms are believed to be an optimum site for the activation of QS, because it is here that natural populations are at their highest cell densities. Many studies in other biofilm-forming bacteria found that biofilm formation and other secreted virulence factors are QS-required. Therefore, the direct relationship between QS and biofilm formation in *B. pseudomallei* also needs further elucidation.

This is the first study demonstrated that when *B. pseudomallei* were grown in condition that induced biofilm formation, they resisted to all antimicrobial agents tested. We quantified the biofilm-forming capacity of 50 soil and clinical *B. pseudomallei* isolates and 5 mutants with their wild types using a microtiter plate assay. Based on the Clinical and Laboratory Standards Institute (CLSI; formerly The National Committee for Clinical Laboratory Standards (NCCLS) and Calgary Biofilm Device (CBD) assay, the *in vitro* susceptibility of planktonic and biofilm cells in each isolate to DOX, CTZ, IMN, and TMP/SMX were compared by evaluating the minimum inhibitory concentration (MIC) of planktonic cells, the MIC of shedding planktonic cells (P-MIC), and the minimal biofilm elimination concentration (MBEC) values. Their LPS phenotypes and LPS pattern profiles were analyzed using SDS-PAGE with silver staining and lastly, the total AHLs in culture supernatants during planktonic and biofilm-formed status were quantified using the bioluminescence assay.

## Materials and Methods

### Bacterial Isolates

Fifty isolates of B. pseudomallei isolated from clinical sources and soils collected from the northeastern endemic region of the country during 1987 to 2001 were used in this study (Table 1). Isolates no. 316a, 316c, 356a, 356c and 979b were kindly provided by Mrs. Vanaporn Wuthiekanun, Mahidol-Oxford Research Unit, Faculty of Tropical Medicine, Mahidol University, Bangkok, Thailand. Capsule, LPS and flagellin defective mutants and their wild types were kindly provided by Prof. Donald E. Woods, Department of Microbiology and Infectious Diseases, University of Calgary Health Sciences Centre, Canada (Table 2). Our 2 biofilm mutants (Table 2) were also included in this study [6]. The mutants were subcultured and grow in Luria-Bertani (LB) agar containing 15 µg/ml tetracycline.

**Table 1:** Detail of 50 *B. pseudomallei* isolates.

Isolates	Source
316a	Blood
316c	Blood
365a	Blood
365c	Blood
979b	Blood
844	Blood
1-20	Blood
1-1217	Blood
1-184	Blood
A2	Blood
A1	Blood
A15	Blood
H1038	Blood
H602	Blood
H63	Blood
H777	Blood
26-2633av	Blood
G12	Pus
3-342	Pus
3-54	Pus
3-82	Pus
3-139	Pus
P87	Pus
P91	Pus
U882b	Pus
G207	Sputum
SP278	Sputum
5-19	Sputum
5-307	Sputum
SP340	Sputum
2-173	Urine
U2704	Urine
U2710	Urine
A20	Skin
A16	Skin
A8	Brain
FL202	Fluid
267	Soil from Northeast region of Thailand
279	Soil from Northeast region of Thailand
354	Soil from Northeast region of Thailand
377	Soil from Northeast region of Thailand
409	Soil from Northeast region of Thailand
429	Soil from Northeast region of Thailand
466	Soil from Northeast region of Thailand
591	Soil from Northeast region of Thailand
705	Soil from Northeast region of Thailand
745	Soil from Northeast region of Thailand
847	Soil from Northeast region of Thailand
877	Soil from Northeast region of Thailand
1219	Soil from Northeast region of Thailand

**Table 2:** Biofilm-forming capacity of *B. pseudomallei* mutants and their wild types.

Isolates	Corrected OD_630 nm_	Biofilm-producing groups
1026b (Wild type) [25]	1.58	Moderate
SR1015 (Capsule-defective mutant) [26]	1.98	Moderate
SRM117 (O-side chain LPS-defective mutant) [25]	2.00	Moderate
MM35 (Flagellin-defective mutant) [25]	0.37	Low
H777 (Wild type) [6]	3.26	High
M10 (Biofilm-defective mutant) [6]	0.16	Very low
M6 (Biofilm-defective mutant) [6]	0.16	Very low

### Biofilm Formation Quantification

A modified microtiter plate test was used to determine the 2-day biofilm-forming capacities of all isolates as previously described [6]. The ability of each isolate to produce biofilm was determined twice in modified Vogel and Bonner's medium (MVBM) which was a chemically defined medium used to facilitate the formation of biofilm [14]. The results reported were the average from two independent experiments. To compare the relative capacity of different isolates to produce biofilm, their OD values were adjusted against that produced by isolate ‘UE5’ of *B. thailandensis* which was randomly selected and used as reference in all experiments. The data was presented as corrected OD_630 nm_ value when compared with the reference isolate. The capability of the bacteria to produce biofilm were arbitrarily classified into 3 groups; low biofilm-producing (corrected OD_630 nm_<1.00), moderate biofilm-producing (corrected OD_630 nm_ = 1.00-3.00) and high biofilm-producing groups (corrected OD_630 nm_>3.00).

### Antimicrobial Susceptibility Testing

#### MIC assay

The minimal inhibitory concentration (MIC) was determined and carried out in 96-well microtiter plates and the interpretation of the results was conducted according to the criteria established by the CLSI (National Committee for Clinical Laboratory Standards NCCLS, 2002). Antimicrobial agents were 2-fold serially diluted in Mueller Hinton broth (MHB) with the final volumes of 50 µl in each well of the plates. For TMP/SMX, MHB containing thymidine phosphorylase (0.2 units/ml) was used. A single colony of each bacteria initially grown on a nutrient agar (NA) plate or LB agar containing 15 µg/ml tetracycline (for mutants) was inoculated into 10 ml of MHB and incubated at 37°C, 200 rpm for 16 h. The culture was further diluted to provide a final inoculum density of 0.5-1×10^5^ CFU/ml in MHB, which was verified by the total viable count. The final inoculum (50 µl) was then added in each well of 96-well microtiter plate. The final concentrations of antimicrobial agents were ranging from 0.12–256 µg/ml for DOX, 0.5–1024 µg/ml for CTZ, 0.12–256 µg/ml for IMN, and 0.06/1.18-128/2432 µg/ml for TMP/SMX. Wells containing only media and culture-free antimicrobial agents were included as negative controls. All samples were run in duplicate. Plates were then incubated at 37°C for 24 h and the MIC was then read. Quality control of the activities of antimicrobial agents was conducted using *Escherichia coli* ATCC 25922 and the MICs for the control strain were within NCCLS limits throughout the study.

#### Antimicrobial preparations for planktonic and biofilm strains

Serial two fold dilutions of each antimicrobial agent in MHB were prepared in the 96-well plates for DOX from 0.25–256 µg/ml, CTZ from 1–1024 µg/ml, IMN from 0.25–256 µg/ml, and TMP/SMX from 0.12/2.37–128/2432 µg/ml with the final test volumes of 200 µl in each well. These antimicrobial plates were used in planktonic and biofilm susceptibility tests.

#### Planktonic and biofilm susceptibility tests within the same strains

The Calgary biofilm device (CBD) (MBEC Biofilms Technology Ltd., Calgary Alberta, Canada) was used for planktonic and biofilm susceptibility testing as described by Ceri *et al.* [15] with slight modifications. The CBD consists of 2 components; the top component forms a lid that has 96 pegs. The pegs are designed to sit in the channels of the bottom component of a standard 96-well plate. Each peg will form the equivalent biofilms [15]. The bacterial biofilm was formed on each pegs in the culture prepared in fresh MVBM with the initial cell concentration of 10^7^ CFU/mL. A final volume (150 µl) of each bacterial culture was placed in each well of 96-well microtiter plate. Medium alone was served as the negative control. The plates were incubated on the rocking platform (Shaker SK-101, HL instruments) at 37°C at approximately 100 rpm for 24 h.

Biofilms formed on the lid of the CBD were then transferred to a standard 96-well plate in which dilutions of the specified antibiotics were prepared. Antimicrobial agent-free wells were also included for growth control by adding only the media. Antimicrobial plates were incubated overnight at 37°C for 24 h, after which the lid was removed and the antimicrobial plates were checked for turbidity in the wells on the microplate reader at 630 nm for determination of P-MIC values. The lid was then rinsed in phosphate-buffered saline, and placed in a second 96-well plate containing MHB. The biofilm was removed from the CBD pegs by sonication for 5 min. A new plate cover was added, and the viability of the biofilm was determined after 24 h of incubation at 37°C by reading the turbidity at 630 nm in a 96-well plate reader for MBEC determinations. The P-MIC is defined as the minimum concentration of antibiotic that inhibits growth of the planktonic bacteria shed from the biofilm during the challenge incubation. The MBEC is defined as the minimum concentration of antibiotic that inhibits regrowth of biofilm bacteria in the recovery media. Clear wells (OD_630 nm_<0.1) are evidence of inhibition.

### Characterization of LPS Phenotype

LPS was extracted from individual *B. pseudomallei* isolates by the proteinase K digestion method [16]. The LPS phenotype of each *B. pseudomallei* isolate was characterized using SDS-PAGE which was carried out in the discontinuous buffer system in a vertical slab gel system [17]. The separating gel contained 15% acrylamide and the stacking gels contained 4% acrylamide. Ten microliters of LPS sample was loaded into each well and electrophoresed. Electrophoresis was carried out at 200 V and LPS bands were then detected with a modified silver stain [18].

### Detection of LPS Alteration in Planktonic and Biofilm Cells

In order to study the role of LPS pattern profile during antimicrobial resistance, the *B. pseudomallei* isolates that had the antimicrobial tests changed from MIC susceptible to P-MIC and MBEC resistance (A15, A16, 5-19, 1-1217, 316c, 705, 844, 3-82, 316a, U882b, A8) or from MIC and MBEC resistance to P-MIC susceptible (365a) were selected for LPS profile analysis. The LPS profile was analyzed by using SDS-PAGE with modified silver stain as described previously [18] in all isolates that mentioned above in planktonic, shedding planktonic and biofilm forms. For planktonic cells, LPS was extracted from overnight broth cultures in 10 ml of MHB and in 10 ml of MVBM using the method as described above. For the isolates that gave resistance in P-MIC values, the planktonic cells that were shed from the pegs were used for LPS extraction. For the biofilm cells, overnight broth cultures of 10 mL in MVMB were subcultured into new fresh MVBM tubes and then incubated statically at 37°C for 2 days. The 2-day biofilm cultures were then used for LPS extraction.

### AHL Assay

AHL production in planktonic and biofilm cells of *B. pseudomallei* was assayed as previously described [19]. The culture supernatant samples were dispensed in aliquots of 100 µl into black 96-well microtiter plates (MicroBiota 1450-405/511, Wallac, Perkin Elmer, MA, USA). Each sample was then mixed with an equal volume of *E. coli* indicator cells (JM109 containing pSB401 [Tet^r^, *luxRluxCDABE*]) which had been grown to an OD of 1 at 600 nm at 30°C in LB broth containing 15 µg/ml tetracycline. The wells containing only medium were also included as negative controls. The plates were then incubated at 30°C for 4 h before bioluminescence counter (Wallac multilabel, PerkinElmer, MA, USA). The amount of AHL was expressed in counts per second (cps) units.

### Statistical Analysis

All data are presented as mean±standard error (SE) and were analyzed using SPSS version 11.5. Comparisons between two and more groups were made by using the Mann-Whitney and Kruskal-Wallis tests. A *P* value of <0.05 was considered statistically significant.

## Results

### Biofilm Productions in *B. pseudomallei* Isolates

The bacterial grown conditions in the microtiter plate test of 50 *B. pseudomallei* isolates demonstrated that each isolate produced a biofilm varying in quantity from one isolate to another (Table 3). The isolate that produced the minimal biofilm was A2 (corrected OD_630 nm_ = 0.59) whereas the highest producing biofilm was U2704 (corrected OD_630 nm_ = 41.91). Most of the soil isolates produced less biofilm than those from clinical isolates although there was no significant difference in the amount of biofilm produced between soil and clinical isolates (means±SE  = 2.39±0.63 and 3.82±1.19 respectively).

**Table 3:** Biofilm-forming capacity of 50 *B. pseudomallei* isolates.

Low biofilm producing group (n = 16)		Moderate biofilm producing group (n = 20)		High biofilm producing group (n = 14)	
Isolates	corrected OD_630 nm_	Isolates	corrected OD_630 nm_	Isolates	corrected OD_630 nm_
267	0.86	409	1.28	279	3.84
354	0.79	705	1.79	377	7.18
429	0.87	844	1.32	U882b	6.98
466	0.95	877	1.36	745	3.63
591	0.84	1-20	1.09	1219	6.96
847	0.87	2-173	1.42	A15	4.90
3-342	0.98	3-82	1.54	A20	3.46
3-54	0.77	1-184	1.31	A8	5.22
5-19	0.85	5-307	1.73	G207	9.88
1-1217	0.84	A1	1.87	H777	3.26
3-139	0.60	FL202	1.00	SP278	4.09
A2	0.59	G12	2.17	U2704	41.91
H1038	0.89	H602	1.15	U2710	15.03
H63	0.88	P87	1.06	A16	13.06
316c	0.92	P91	1.70		
979b	0.63	SP340	2.67		
		316a	1.58		
		365a	1.13		
		365c	1.56		
		26-2633av	1.15		
Mean±SE	0.82±0.12	Mean±SE	1.49±0.09	Mean±SE	9.24±2.69

For the mutants and their wild types, the biofilm-forming capacity of the capsule-defective mutant, SR1015 and O-side chain LPS-defective mutant, SRM117, were slightly more than their 1026b wild type (Table 2). This indicated that the capsule and O-side chains of LPS do not involve biofilm formation by this bacterial species. Conversely, the biofilm-forming capacity of the flagellin-defective mutant, MM35, was markedly reduced when compared with its wild type. For biofilm defective mutants, both M10 and M6 were found to have very low biofilm production as expected since they were biofilm-defective mutants.

### Susceptibility of Planktonic and Biofilm Cells of *B. pseudomallei* to Antimicrobial Agents

**Figure 1:**
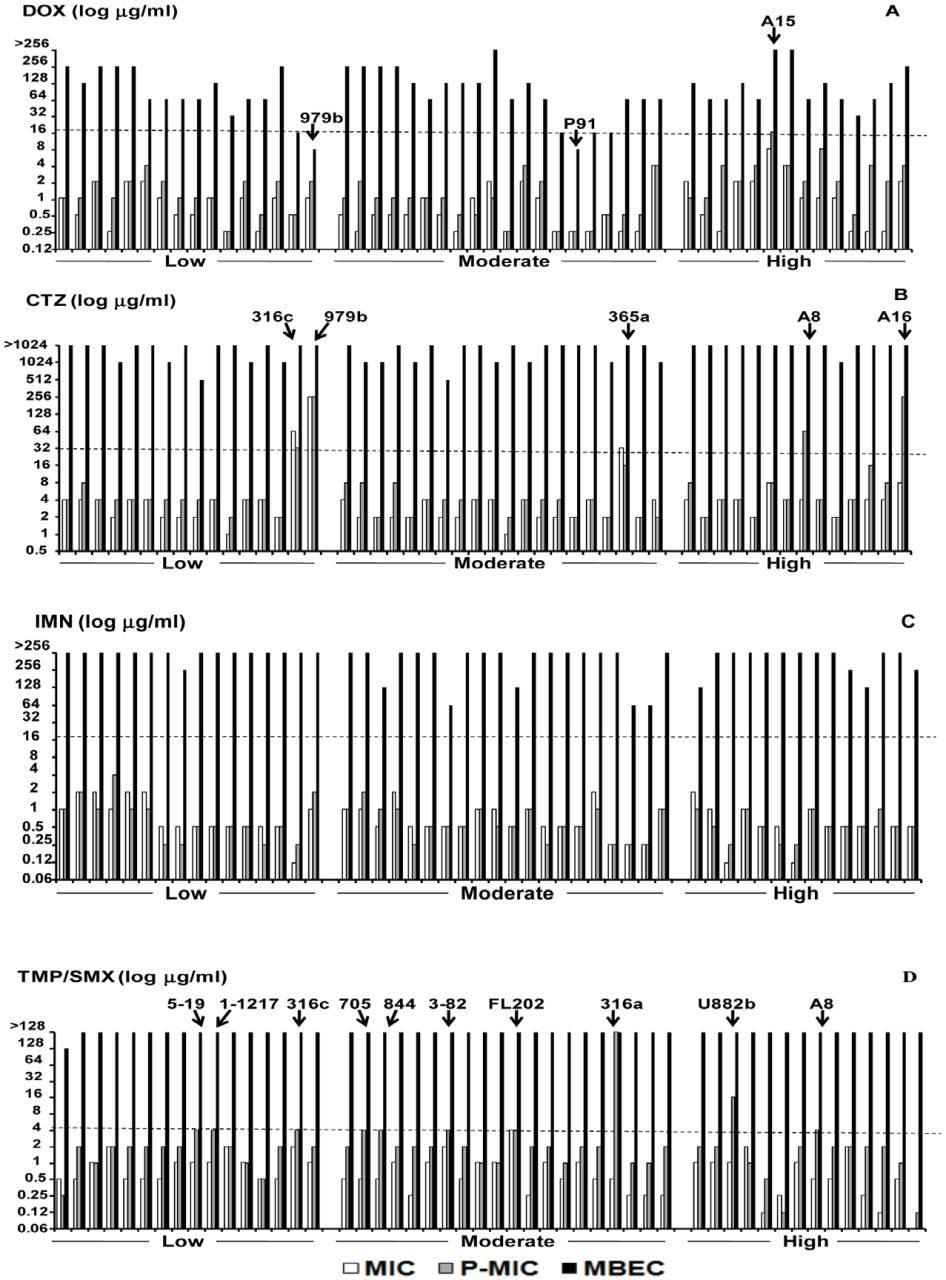
The response of *B. pseudomallei* planktonic and biofilm cells to antimicrobial agents. Susceptibility of planktonic and biofilm cells of *B. pseudomallei* isolates to doxycycline (DOX; A), ceftazidime (CTZ; B), imipenem (IMN; C) and trimethoprim/sulfamethoxazole (TMP/SMX; D) were shown. The cut off (---) indicates the resistant lines.

The concentrations of four antimicrobial agents required to inhibit planktonic cells (MIC), shedding planktonic cells (P-MIC) and those required to kill biofilm bacteria (MBEC) of low, moderate and high biofilm producing *B. pseudomallei* isolates are shown in Figure 1. Most of the MICs and P-MICs of all antimicrobial agents used gave similar results. From the MIC and P-MIC results, all isolates were susceptible to IMN (Figure 1C). Most isolates except A15 were susceptible to DOX (Figure 1A). A15 was susceptible by MIC but resistant by P-MIC. Five clinical isolates, 316c, 365a, 979b and A8, A16 were resistant to CTZ when determined either by MIC and P-MIC respectively (Figure 1B). The remaining isolates were susceptible while 10 isolates, FL202, 5-19, 1-1217, 316c, 705, 844, 3-82, 316a, U882b and A8 were resistant to TMP/SMX as determined by P-MIC values (Figure 1D). When these bacteria were induced to form biofilm, they were highly resistant (MBEC results) to all antimicrobial agents tested. Only 2 biofilm bacteria, 979b and P91, were found to be susceptible to DOX.

Most of the planktonic cells of all 3 biofilm-producing groups, low, moderate, and high, were susceptible to all antimicrobial agents tested. When the MIC, P-MIC, and MBEC values among three biofilm-producing groups were compared, there were no significant differences in antimicrobial resistance. These data also indicated that, when bacteria were induced to form biofilms, most of them exhibited resistance to antimicrobial agents as shown in the MBEC values regardless of their biofilm-producing capacity.

**Figure 2:**
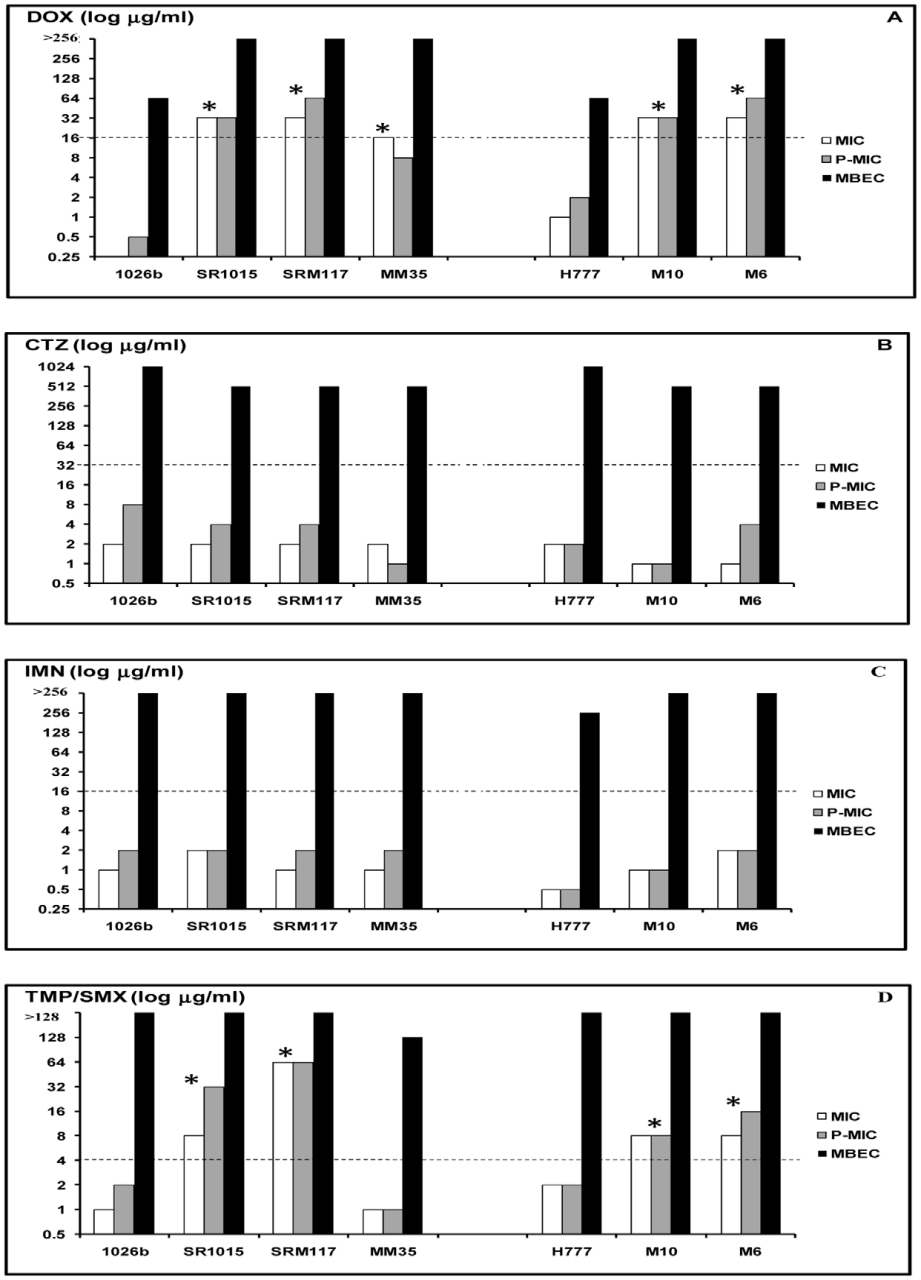
The response of *B. pseudomallei* mutants and their wild type to antimicrobial agents. Susceptibility of *B. pseudomallei* mutants and their wild types to doxycycline (DOX; A), ceftazidime (CTZ; B), imipenem (IMN; C), and trimethoprim/sulfamethoxazole (TMP/SMX; D) were shown. The cut off (---) indicates resistant lines. The astericks (*) refer to resistant strains.

From the MIC and P-MIC results, the wild type isolates, 1026b and H777, were susceptible to all antimicrobial agents (Figure 2) whereas the biofilm cells of these 2 isolates were resistant. All mutants except MM35 were resistant to DOX and TMP/SMX (Figure 2A and 2D). None of the mutants were resistant to CTZ and IMN (Figure 2B and 2C). When all mutants were induced to form biofilms, they were highly resistant to all antimicrobial agents even in biofilm-defective mutants, M10 and M6 (Figure 2). Since all mutants were constructed using transposon containing the Tet^R^ gene, they were therefore found to be resistant to DOX (Figure 2A).

### LPS Phenotype of *B. pseudomallei* during Biofilm Production and Antimicrobial Resistance

Of 50 *B. pseudomallei* isolates, 39/50 or 78% possessed smooth type A and 14% or 7/50 possessed smooth type B LPS. The remaining 4/50 or 8% without a ladder appearance possessed the rough type. For mutants, all strains exhibited the LPS phenotype similar to their wild types with the exception of SRM117 which possessed the rough type phenotype since it lacked O-side chain moiety in the LPS structure (data not shown). The smooth type B and rough type LPS *B. pseudomallei* isolates appeared to have a significantly higher capacity to produce biofilm than the smooth type A (*P*<0.05). The corrected OD_630 nm_ values (mean±SE) obtained from smooth type B and rough type LPS *B. pseudomallei* were 10.37±5.56 and 7.36±2.55, while that value of smooth type A *B. pseudomallei* was 1.81±0.26. No significant difference of biofilm producing capacity was observed between the smooth type B and rough type LPS *B. pseudomallei* isolates.

**Figure 3:**
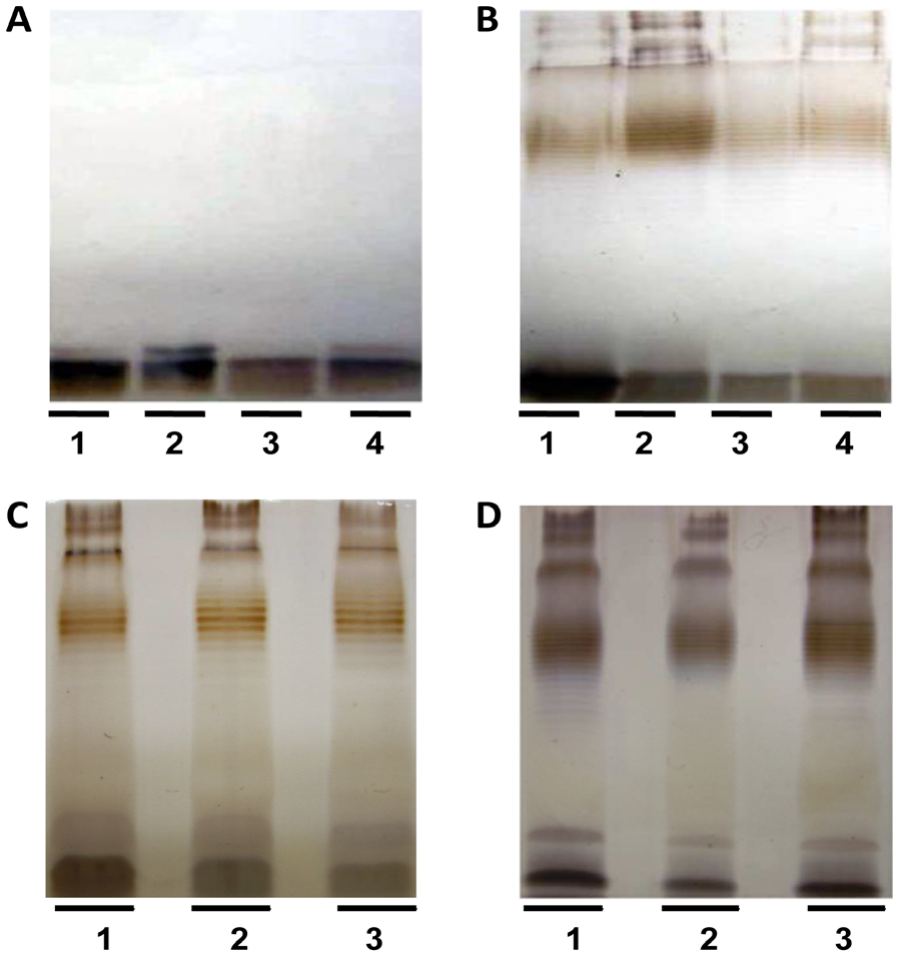
LPS profiles of planktonic, shedding planktonic and biofilm cells of *B. pseudomallei* isolates during changing their antimicrobial susceptibility. LPS profiles of rough type isolate, A16 (Panel A) and smooth type A LPS isolate, 5-19, (Panel B), during planktonic status cultured in MHB medium in lane 1, MVBM medium in lane 2, shedding planktonic status in lane 3, and 2-day biofilm-formed status in lane 4. The LPS profile of smooth type B isolate, U882b (Panel C) obtained from its planktonic status in MVBM medium (lane 1), shedding planktonic (lane 2), and 2-day biofilm-formed status (lane 3). (Panel D) LPS profile of 365a isolate which was resistant to CTZ during planktonic status cultured in MHB medium (lane 1) and MVBM medium (lane 2), and 2-day biofilm-formed status (lane 3).

The LPS patterns in figure 3 demonstrated the examples of 4 from 12 isolates which all of them showed no alteration of LPS phenotype when the bacteria changed from MIC susceptible to P-MIC and MBEC resistances (A16, 5-19 and U882b) or MIC and MBEC resistances to P-MIC susceptible (365a) (Figure 2).

### AHL Synthesis in Planktonic and Biofilm Cells of *B. pseudomallei* Isolates

**Figure 4:**
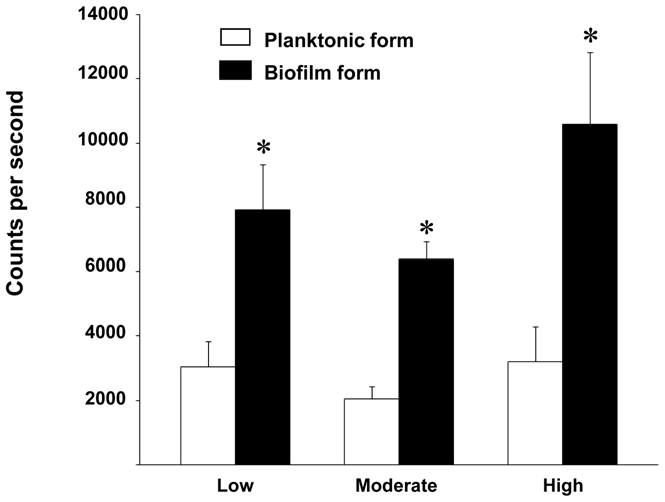
AHL synthesis from planktonic and biofilm-formed *B. pseudomallei*. The 50 *B. pseudomallei* isolates were divided into 3 groups: low (n = 16), moderate (n = 20) and high (n = 14) biofilm producing isolates. The amounts of AHL were determined in culture supernatants of the planktonic (open bar) and 2-day biofilm-formed (solid bar) by using a bioluminescence assay with the reporter strain (*E. coli* JM 109 containing pSB401). Data were expressed as counts per second and shown as mean±SE of each groups. The asterisks (*) represent a significant difference (p<0.05).

The amount of AHL in the culture supernatants of 2-day biofilm cells in all biofilm-producing groups were significantly higher than those of planktonic cells (*P*<0.05) (Figure 4). The amount of AHL, however, did not correlate with the biofilm producing capability of *B. pseudomallei*.

## Discussion

Melioidosis is still a serious infectious disease that requires a long course of antimicrobial therapy such as intravenous CTZ or carbapenems for at least 10 days, followed by oral antimicrobial agents, DOX, TMP/SMX or combination therapy for at least 12 weeks [20]. Relapse of the disease is still common despite adequate antimicrobial therapy [8]. *B. pseudomallei* was reported to form biofilm both in laboratory media and in animal model [5]. The role of biofilms in protecting *B. pseudomallei* against antimicrobial agents has been reported in one study using a modified Robbins device [21]. The biofilm cells in their study were still viable after 24 h of antimicrobial exposure, with up to 200 times of the MIC of planktonic cells. However, only one *B. pseudomallei* isolate was tested against CTZ and TMP-SMX. Moreover, it was not the direct comparison between the planktonic and biofilm cells of the same *B. pseudomallei* isolate. The used of the Calgary Biofilm Device, MBEC™ device [15], in our study can directly compare the antimicrobial resistance of the bacterial sloughed or shed from the surface of the readily formed biofilm and serve as the inoculum for P-MIC and MBEC determinations. Although the P-MIC values obtained using the MBEC™ device are similar to those obtained using the NCCLS procedure [15], a difference of planktonic MIC values between 1 to 3 dilutions when obtained from both assays was observed. Therefore, in this study the NCCLS assay for planktonic MIC determination using the standard microdilution was performed in parallel with the MBEC™ assay. In the mutant study, the biofilm-forming capacity of capsule-defective SR1015 and O-side chain LPS-defective SRM117 mutants was slightly higher than their 1026b wild type while the MM35 flagellin-defective mutant produced the lowest biofilm quantity. This result suggested that flagellin, but not capsule nor LPS, was required for biofilm formation. The flagellin may be required for adherence of the planktonic cells to the surface, since it has been reported that flagella and twitching motility were necessary in the development of *Pseudomonas aeruginosa* biofilms formation [22].

Most of the 50 *B. pseudomallei* planktonic cells were susceptible to all antimicrobial agents used in this study. This confirmed previous reports on the *in vitro* susceptibility to antimicrobial agents of *B. pseudomallei* isolates with the current recommendations of these drugs for the treatment of melioidosis [23]. The MIC and P-MIC values were within the susceptible limits but varied in their ranges compared to the previous report [23]. There were some isolates that gave different results. This dissimilarity might be due to the different of methods since MIC is antibiotic efficacy tested against bacteria from seeding whereas P-MIC is the antibiotic efficacy tested against the planktonic bacteria shed from the biofilm. Among antimicrobial agents used, IMN showed the greatest activity with extremely low MIC values and the planktonic cells of all isolates were susceptible to this drug. This suggested the use of IMN as an alternative to CTZ in the treatment of disseminated or severe melioidosis or in case of resistance to CTZ. In contrast, the MIC of one isolate (2%, 1/50), FL202, was resistant to TMP/SMX, and 3 isolates (6%, 3/50), 316c, 365a and 979b, were resistant to CTZ (Figure 1). The primary CTZ resistance in these 3 isolates has been proved to result from their own β-lactamase-based mechanisms for CTZ [24]. The resistance to DOX of all planktonic mutants was due to transposon-carrying tetracycline-resistant genes that be inserted into the wild type strain in the mutant construction process [25], [26]. It was also observed that these planktonic mutants were likewise resistant to TMP/SMX, except for the flagellin-defective mutant, MM35. The underlying mechanism of this finding is still unknown and may possibly be due to the random insertion of tranposons and affects the expression of resistance genes for this antimicrobial agent.

The biofilm cells *B. pseudomallei* were shown to be markedly more resistant to antimicrobial agents than the corresponding planktonic cells within the same isolate, consistent with the results obtained using the same methods in other biofilm bacteria [27]. Several mechanisms were proposed in the role of how the biofilm affects antimicrobial resistance including the antibiotic diffusion limitations of the biofilm matrix and the heterogeneity of growth rates within the biofilm [28]. The difference of bacterial density throughout the biofilm determines gradients of nutrients and oxygen availability within biofilm structure, results in differences of metabolic activity among bacteria that could restrict the growth of bacteria [29]. Because most antimicrobial agents primarily target metabolically active cells, the slow growth rate and metabolic heterogeneity in biofilm cells have been proposed to contribute resistance to antimicrobial agents, particularly the β-lactams [30], [31]. Our result demonstrated that high resistance still occurred even in our two biofilm-defective mutants. The mechanism of biofilm as barrier in antimicrobial diffusion is therefore unlikely. The resistant mechanism might not be due to the biofilm formation by itself but because of the conditions that induced the biofilm formation. Because the biofilm formation is multifactorial process, when the biofilm gene operon was induced, it might also induce some other set of genes or some other mechanisms which responsible for drug resistance. No correlation between amount of biofilm and AHL productions in our 50 *B. pseudomallei* isolates and antimicrobial resistance were another evidence to support our hypothesis. Moreover, biofilm formation was reported to enhance the rate of mutability due to the accumulation of DNA damage [32]. This may enhance the opportunity to drive the selection of antibiotic-resistant organisms [32]. CTZ and IMN, the two β-lactams antibiotics, were totally ineffective in killing *B. pseudomallei* biofilm cells. The explanation of this resistance may be due to slow growth rate. The studies done in *P. aeruginosa* demonstrated that β-lactams and tetracycline showed poor bactericidal activity against non- or slow-growing cells of *P. aeruginosa* biofilm producers [33], [34]. Alternatively, CTZ could be destroyed by the production of inactivating enzymes such as β-lactamase that accumulated within the glycocalyx of the *P. aeruginosa* biofilm [35], [36]. The resistance mechanism to TMP/SMX in *B. pseudomallei* biofilm organisms is still unclear, although it has been documented as being due to the production of a different dihydrofolate reductase enzyme in planktonic-resistant cells [37].

LPS has been shown to be important for the resistance of the bacteria to antimicrobial agents due to its barrier to antimicrobial penetration [38]. The nature of this barrier is associated with changes in the composition and phenotype of LPS in different bacterial species tested [39], [40], [41]. For *B. pseudomallei*, the results showed that the LPS extracted from the planktonic, biofilm and shedding cell of the same organisms gave the same LPS pattern profiles. Moreover, the difference in LPS phenotypes was not correlated with MIC, P-MIC, or MBEC. This indicated that the composition or phenotype of LPS was not altered while forming biofilms and was not correlated with the antimicrobial resistance mechanism in *B. pseudomallei*.

Within the genus *Burkholderia*, particularly in some strains of *B. cenocepacia* and *B. cepacia*, their quorum sensing (QS) systems positively regulated expression of swarming motility and biofilm formation [42]. These QS were not involved in the regulation of initial cell attachment but rather controlled the maturation of the biofilm. From our results, a significant difference in the AHLs was detected in culture supernatants of planktonic when compared with biofilm-formed cells. This indirectly suggests that these isolates utilized AHLs to regulate the high biofilm production. Nevertheless, the highly resistant biofilm cells were not AHL dependent since there were no differences in MBEC values in the different of AHLs production (data not shown). This finding is similar to what has been observed in *B. cenocepacia* biofilms [43]. Moreover, the production of AHLs was not correlated with the phenotypes of LPS and the biofilm-forming capacity. It should be kept in mind that the biofilm AHLs detected in culture supernatants are not the total amounts. These levels of AHLs could not be extrapolated to the presence of AHLs within the biofilms themselves. Although it has been demonstrated that *B. pseudomallei* could produce numerous AHLs [13], [44], [45], the one that play a direct role in biofilm production is still unknown. The clinical importance of AHL-mediated biofilm formation and the directly regulated-QS circuits involved in *B. pseudomalli* are the subject of an on-going investigation.

In conclusion, the conditions to induce *B. pseudomallei* biofilm formation were proven to be highly resistant to all antimicrobial agents tested when compared to the corresponding planktonic cells of the same isolates. The barrier of biofilm in preventing the drug penetration was proved to be unlikely and the biofilm mutant which resistant to all drugs after they were induced to form biofilm raised a possible mechanism of drug resistance that may be up-regulated together with the stimulation of biofilm formation. The biofilm cells, if present *in vivo* during bacterial infection might contribute to a long persistence of the bacteria and consequence to the high relapse of the disease. The contribution of AHL in their higher biofilm production was proposed but LPS phenotypes did not change during the antimicrobial resistance or biofilm production. The understanding of this phenomenon will lead insights in the control of biofilm formations and prevention of relapse in melioidosis.
